# Non-Standard Electrode Placement Strategies for ECG Signal Acquisition

**DOI:** 10.3390/s22239351

**Published:** 2022-12-01

**Authors:** Margus Metshein, Andrei Krivošei, Anar Abdullayev, Paul Annus, Olev Märtens

**Affiliations:** Thomas Johann Seebeck Department of Electronics, Tallinn University of Technology, Ehitajate tee 5, 19086 Tallinn, Estonia

**Keywords:** electrocardiography, electrode placement strategy, impedance cardiography, QRS complex, RR interval, signal quality assessment, T wave

## Abstract

Background: Wearable technologies for monitoring cardiovascular parameters, including electrocardiography (ECG) and impedance cardiography (ICG), propose a challenging research subject. The expectancy for wearable devices to be unobtrusive and miniaturized sets a goal to develop smarter devices and better methods for signal acquisition, processing, and decision-making. Methods: In this work, non-standard electrode placement configurations (EPC) on the thoracic area and single arm were experimented for ECG signal acquisition. The locations were selected for joint acquisition of ECG and ICG, targeted to suitability for integrating into wearable devices. The methodology for comparing the detected signals of ECG was developed, presented, and applied to determine the R, S, and T waves and RR interval. An algorithm was proposed to distinguish the R waves in the case of large T waves. Results: Results show the feasibility of using non-standard EPCs, manifesting in recognizable signal waveforms with reasonable quality for post-processing. A considerably lower median sensitivity of R wave was verified (27.3%) compared with T wave (49%) and S wave (44.9%) throughout the used data. The proposed algorithm for distinguishing R wave from large T wave shows satisfactory results. Conclusions: The most suitable non-standard locations for ECG monitoring in conjunction with ICG were determined and proposed.

## 1. Introduction

Electrocardiography (ECG) is an essential technique to monitor the health of the heart [1]. The technique relies on the electrical activity, generated by the sinus node (i.e., sinoatrial node) that is located in the wall of the right atrium. The sinus node represents a natural controller of the heart, coordinating certain sources of pumping function [2]. ECG is widely used in medicine—often for full-time supervision of the cardiac condition in clinical medicine but also for pathological purposes.

**Figure 1:**
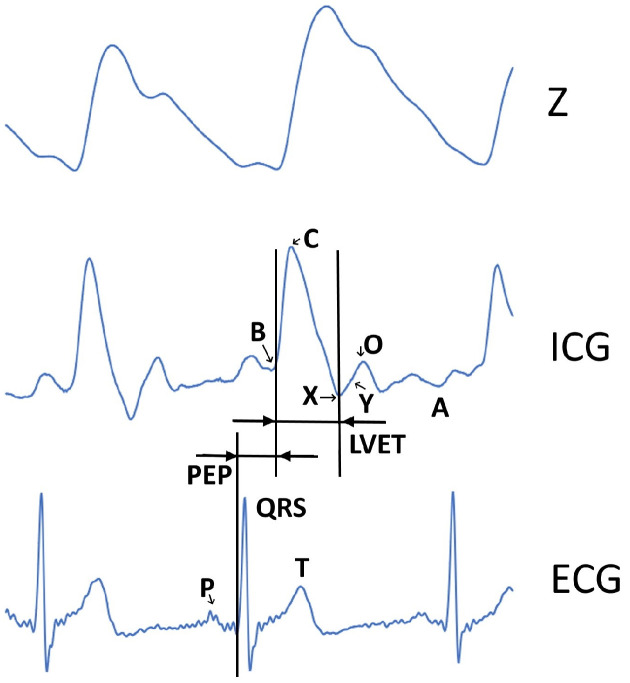
Typical patterns of the impedance Z, the resulting impedance cardiography (ICG) waveform (with the marked hemodynamic fiducial points B, C, X, Y, O, and A), and electrocardiography (ECG) waveform (with the waves P-QRS-T and relevant cardiac time intervals left ventricular ejection time (LVET) and pre-ejection time (PEP)) (from top to bottom).

Electrocardiogram, the result of ECG, is a graph that presents a signal of distinguishable pattern as voltage in time scale. The unique pattern with graphical deflections, from which the QRS complex is most widely known, corresponds to the depolarization, i.e., activation of the ventricles (example signal waveform of the practically acquired ECG can be seen in Figure 1). In addition to QRS complex, the following waves are also known on the electrocardiogram (Figure 1):

- P wave—represents the depolarization of atriums;
- T wave—represents the repolarization of ventricles.

Similarly, certain fiducial points (B, C, X, Y, O, A) are defined on the signal of impedance cardiography (ICG), representing certain cardiac events and time intervals (left ventricular ejection time (LVET), pre-ejection period (PEP)) (Figure 1)).

To measure ECG, electrodes (typically commercial wet gel Ag/AgCl electrodes) are attached to predetermined locations on the chest, arms, and (or) legs. More specifically, the locations depend on the selected electrode (or lead) system, which is developed to access detailed data of a certain cardiac process or the activity of the sinus node. The most used system is the 12-lead system, followed by the 5-lead and the standard 3-lead system [3].

ECG is becoming increasingly popular in personal medicine through rapid progress in the world of personal wearable devices (such as smartwatches) [4]. The problem that has been hindering the development of such gadgets for ECG monitoring is the need to attach the electrodes close to the source, i.e., the location of the sinus node. In simple words, depending on the selected number of leads, the location of the heart is expected to remain in the area close to the electrodes. This is the reason why, e.g., in smartphone- (or watch-) related applications, for recording the ECG, the finger of the other hand has to contact a certain conductive pad of the device [5]. In principle, such a solution relies on the signal that is acquired from lead I. However, the convenience of such a solution is questionable, not enabling adequate, unnoticeable, continuous monitoring. One can imagine that when the monitoring of ECG could be performed regionally on a restricted area of the body surface, with some gadget such as a wrist-watch, the result would be a game-changer.

The feasibility of acquiring ECG from the arm and wrist was confirmed almost 15 years ago [6]. In several publications, the accessibility of electrocardiograms on the upper arm has been demonstrated [7,8]. In [9], a variety of electrode locations were utilized on a single arm, including the wrist; the results back then were promising, showing that after denoising, in 88 % (*n* = 32) of the cases, the electrocardiogram was usable. On top of that, solutions for the electrodes and methodologies have been built (e.g., [10]).

The farther away from the sinus node, the weaker the signal of ECG due to the attenuation and impedance of conductive biological matter (blood, tissues, etc.). Nevertheless, it has been shown in scientific literature that the ECG can be monitored regionally [6,7,10].

However, when the intention is to design a wearable device, the non-standard electrode placements can cover other body areas as well. Moreover, a variety of sensors are often simultaneously used to measure cardiovascular system parameters besides ECG [11]—such as ICG, photoplethysmography (PPG), inertial measurement unit (IMU), etc. For picking up the signal of impedance for extracting the impedance cardiogram, a variety of electrode placement strategies (EPC) have been shown to give promising results [12]. The design of a wearable device, where the sensors co-employ the same locations, is expected to be more compact [13]. However, trade-offs are needed through applying possibly non-standard locations for different sensors.

The attempts to gain ECG from non-standard locations of the human body have been reported in the literature before, especially in the light of wearable devices. For example, a wearable-device-targeted research was conducted, describing the monitoring of ECG with a single lead from the chest, back, shoulder, and hip [14]. A similar approach, implemented as a smart chair, to acquire a single-lead ECG signal from the chest and back from non-standard locations is described in [15]. Conversely, a reduced area I or III lead gadget can be attached to the chest [16,17].

An important aspect here is the noise, causing an increased signal-to-noise ratio (SNR), originating from a variety of sources. Here, the signal denoising techniques are expected to assist (an excellent review is given in [18]). However, to perform signal quality assessment (SQA), i.e., signal categorization based on different features (e.g., very generally, fiducial, and non-fiducial), the signal is expected to be denoised. As follows, certain signal parameters are analyzed to eliminate the unusable ones or classify them based on the desired features.

This paper focuses on the acquisition options of ECG signal from non-standard locations, dictated by the measurement strategies of ICG. Classically, ICG is gained from the thorax (starting with the pioneering method proposed by Sramek [19]). However, with the spread of wearable technologies, the electrode strategies have changed to be more compact or focused on certain body areas (from which the arm has provided promising results [20]). This research combines the monitoring of ECG and ICG by following the principles of compactness and miniaturization in contact between the medium and wearable device.

The research is performed in the frame of developing low-energy-consumption intelligence hardware for biomedical sensors with the goal of enhancing the wearable wireless monitoring concept. The focus is placed on the signals of ICG and ECG, presumably not with the highest quality. The possible novel regional (e.g., arm) and non-standard EPCs are considered in the light of jointly acquired signals of ICG for smart innovative Holter monitor-like solutions. The target is to develop a “near-sensor computing” approach, relying on non-uniformly sampled data from level-crossing analog-to-digital converters, implemented through an analog algorithm, circuit, and system development.

The signals of ECG, acquired from a variety of non-standard locations (including single arm), are assessed to select the best ones. Comparison of the most suitable EPCs for ECG signal acquisition and the comparison to the EPCs for ICG signal acquisition (published by the authors in [12]) is performed. Suggestions for EPCs and the design of wearable devices (for both hardware and software) for the joint acquisition of the signals of ECG and ICG are given.

For evaluating the signals of ECG, an SQA approach, relying on the presence of R, T, and S waves is applied for practical reasons. An approach for distinguishing R wave in the case of large T wave is presented and applied on the ECG signals acquired by the non-standard EPCs—comprising a novel approach. The reason for proposing a solution for overcoming the large T waves and not other morphology-related deviations (such as short QT, deflected P wave, etc.), is the absence of these issues in the utilized data set.

The current paper is structured as follows. Section 2 introduces the relevant SQA methods for ECG signal analysis. In Section 3, the devices and the methods of acquisition of the utilized signals and proposed SQA are described. The results are presented in Section 4, and are discussed and concluded in Section 5, Section 6 and Section 7, respectively.

## 2. Signal Quality Assessment for ECG Signals

In this section, firstly, a concise review of the methods applied in literature for performing SQA of ECG signals is presented. Secondly, the method used in current paper to perform SQA of ECG signals acquired from non-standard EPCs is described. Thirdly, an approach is proposed and described to solve an appearing problem with the utilized measurement data—distinguishing R waves from large T waves.

### 2.1. Overview and Perspective

A continual issue with all kinds of bio-originated signals is the noise. Main types of noises that affect the signal of ECG are power line interference, electrical activity of muscles and respiratory-activity-related (or other volume change) modulation of the signal (i.e., the baseline drift) [21,22]. However, the additional (and maybe more severe) deterioration of SNR, especially in the case of wearable devices, may be caused by the following [23]:

- Uncertain contact between the electrode(s) and skin, causing the saturation and disconcerting peaks in acquired electrocardiogram;
- Motion artifact (MA)-induced interference, caused by the shift of electrode(s) relative to the skin surface causing the impedance of the skin-to-electrode interface to vary;
- Wearable device hardware related artifacts.

In literature, the SQA methods are applied in three consecutive stages [22]:

- Pre-processing, i.e., noise removal through filtering;
- Feature extraction, i.e., extraction of certain ECG signal features;
- Classification, i.e., labeling, resulting as designation of signals into quality categories.

All these stages are equally important, starting from the first one. The noisy and disturbed signals cause false alarms—for example, in cardiac arrhythmia detection—due to the inability to determine the actual ECG waveform [24]. The solution can be hidden in pre-selection of usable periods of the signal [22]. A variety of techniques and approaches exist such as moving average filters [25], polynomial filters [26], discrete wavelet transform [27], Bayesian filter [28], etc.

The SQA approach, i.e., the definition of certain signal quality indexes, has gained large attention in scientific literature up to today [29,30]. A variety of SQA approaches have been proposed; the majority include classifying the signals to fulfill certain conditions—in trivial cases: acceptable or unacceptable (e.g., [31,32]. However, approaches where more conditions have been proposed exist (e.g., [33]). Today, the machine learning and heuristic-classifiers-based approaches for SQA of ECG signals constitute the vast amount of research literature [22,32].

A wide variety of SQA approaches have been published which can generally be listed as follows [22]:

- Fiducial points detection SQA approaches;
- Non-fiducial properties detection SQA approaches;
- Filtering-based SQA approaches.

Fiducial points denote certain moments or time intervals on ECG signal waveform, i.e., they are certain features of human body and its systems. Non-fiducial properties are the features of the signal, which are found during signal conditioning in time and frequency domain (but may also be statistical).

### 2.2. The Method—Signal Quality Assessment

A trivial fiducial point detection based SQA approach is applied in present research to select the most suitable non-standard EPCs for monitoring the ECG. The comprehensive target is to detect the QRS complex based on corresponding fiducial points (omitting currently the Q wave), supplied additionally with R wave determination from large T wave. However, the beat-to-beat interval is also considered together with the respective ratio of the maximum beat-to-beat interval to the minimum beat-to-beat interval. The selected features of assessing the signal of ECG in current research are listed subsequently.

1. Peak value (global positive extremum) of R wave (A_Rwave_);
2. Peak value of T wave (A_Twave_);
3. Peak value (global negative extremum) of S wave (A_Swave_);
4. Interval between two R waves (T_RRint_);
5. Ratio of maximum RR interval to minimum R interval (R_RRintmaxmin_).

To support the determination of the most suitable EPCs, the following parameter was calculated based on the selected features of ECG signal.

- The ratio of difference between the minimum and maximum peak values to the maximum values of R, S, and T waves (relative peak amplitude).

**Figure 2:**
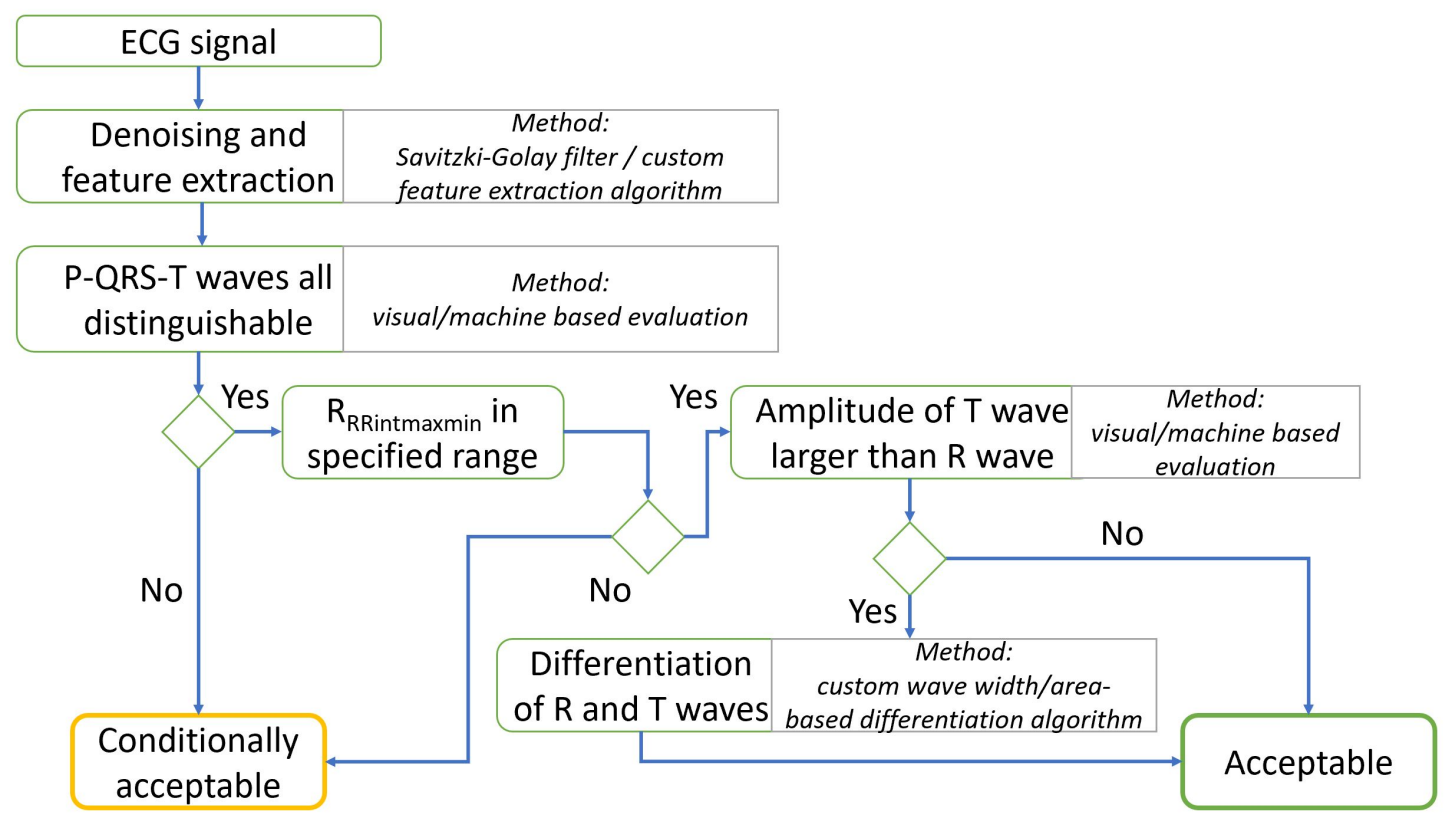
The applied decision tree for categorization of the signals of ECG.

The signals were classified simplistically into two categories of “conditionally acceptable” or “acceptable”, which are not much used in specialized literature. The standard trivial approach is to divide the signals into either good or bad [29,34,35]. However, in the case of this work, the analyzed signals provide at minimum the recognizable presence of R wave, which is enough to determine the RR interval (e.g., HR) but, in the majority of cases, also other waves. The decision tree can be seen in Figure 2.

The SQA of the signal of ECG is preceded by the signal denoising through, e.g., Savitzky–Golay low-pass filtering. Then, feature extraction is performed, e.g., in the form of fiducial point (R, S, and T waves) detection. On top of that, three rules are followed to categorized the signals:

1. All chosen waves are visible, i.e., the result of feature extraction—the signal is conditionally acceptable and usable even if some waves are indistinguishable.
2. Heart rate (HR) between 40 and 180 beats per minute (bpm) [29]. While the normal adult HR is 60–100 bpm, the possible range is much higher, depending on several physiological and medical factors.
3. Amplitude of the T wave larger than R wave. As a medical condition, this pathology refers to hyperkalemia [36] or transmural ischemia [37]; however, such a result may appear also in the case of non-standard EPCs.

If the conditions apply, the signal can be accepted.

### 2.3. The Method—Distinguishing R Wave from Large T Wave

In the case where the amplitude of a T wave is comparable or larger than the amplitude of an R wave, an approach for distinguishing them is needed. We propose a novel peak properties comparison-based approach to distinguish between the R and T waves.

The morphology of the T wave can vary, depending on pathological aspects but also on the measurement methodology. Four classical types of changes in T waves have been identified in literature [38]: large T wave (1), inverted T wave (2), flattened T wave (3), and biphasic T wave (4). However, depending on the chosen lead for measuring the ECG, the reason for deviation of the morphology of T waves from the regular shape can depend on the chosen EPC [39]. This may appear in the case of wearable-device-targeted designs.

A variety of approaches have been proposed in literature to determine the properties of T wave, especially in the context of measuring the QT interval. QT interval is an important variable in following the status of the patient on potentially pro-arrhythmic drugs [40]. Still, the T wave itself proposes a challenge, which has been proposed to be solved, among others, through the application of wavelet transform [41], artificial neural networks [42], signal morphology [43], ensemble averaging [44], and derivatives of the signal [45].

**Figure 3:**
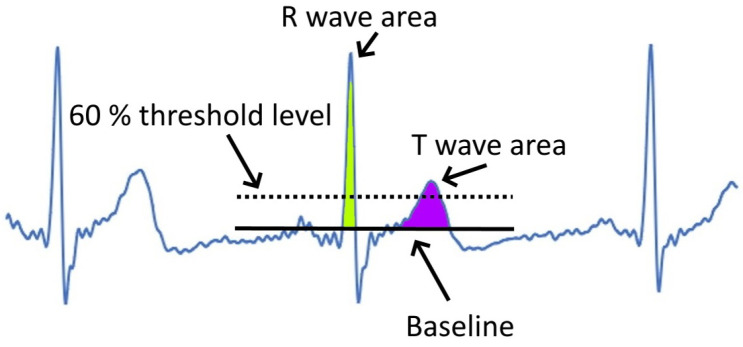
Illustration of two approaches for distinguishing the R and T waves on the signal of ECG (blue line) based on the width and area ((painted green for T and purple for T wave) of the wave.

Specifically, we propose two approaches: determination of the average width of R and T waves at 60% threshold level (1). Secondly, the calculation of the (pseudo) area of the peak, which in the case of R wave is presumably smaller (2). A depiction of the method can be seen in Figure 3.

The area of the peak can be calculated simply as

(1) Area=s×V,

where *s* is time in seconds and *V* is voltage.

In our approach, we use three windows next to each other where the point under test (R wave) is assumed to be in the middle window. In other words, we try to check that the middle window contains the R wave and use for this the validation neighbors’ window parameters as well. We scale the windows by the maximum value of all three windows and perform another calculation for each window; we find the ratio ($RA_{AreaToAmpl}$) of area (Area) of each window to its amplitude (maximum value) based on

(2) $$RA_{AreaToAmpl}=Area/V,$$

In the area calculation, we use only the positive part above the baseline of the signal in the window (Figure 3).

For the validation of the R wave in the middle window, we provide the next conditions:

(3) $$\begin{array}{c} \left\{\begin{array}{cc} RA_{AreaToAmpl}\;\left[middle\right] & <RA_{AreaToAmpl}\;\left[right\right]\\ Ampl\;[left] & <0.5\\ Ampl\;[middle] & >=0.5 \end{array}\right. \end{array}$$

The area-based approaches for determining the T wave have been proposed in literature [46,47]. Typically, these approaches are based on a single sliding window function, which is an effective way for variety of signals. Further, these are effective for wearable, battery operated devices, where the simplicity of the approach is appreciated. The signal in the case of personal gadget is individual, and can be individualized through calibration. Our two-stage approach is expected to be enough in the case of the targets of the current research.

## 3. Materials and Methods

In this section, the measurement devices and utilized methodology for acquiring the signal of ECG by using the non-standard EPCs is described.

### 3.1. Measurement Devices

The signals were acquired using the impedance spectroscope HF2IS, which was supplied by the transimpedance amplifier HF2TA (Zurich Instruments AG (Zurich, Switzerland)) [48]. For the acquisition of the signal of ECG, the custom-made bipolar 3-lead ECG monitor, based on the single-lead heart rate monitor front-end AD8232ACPZ (Analog Devices (Wilmington, MA, USA)) was utilized. Standard monitoring electrodes of type 2228 (3M (Maplewood, MN, USA)) were used to achieve electrical contact to the body.

### 3.2. The Method—Measurement Data and Its Acquisition

**Figure 4:**
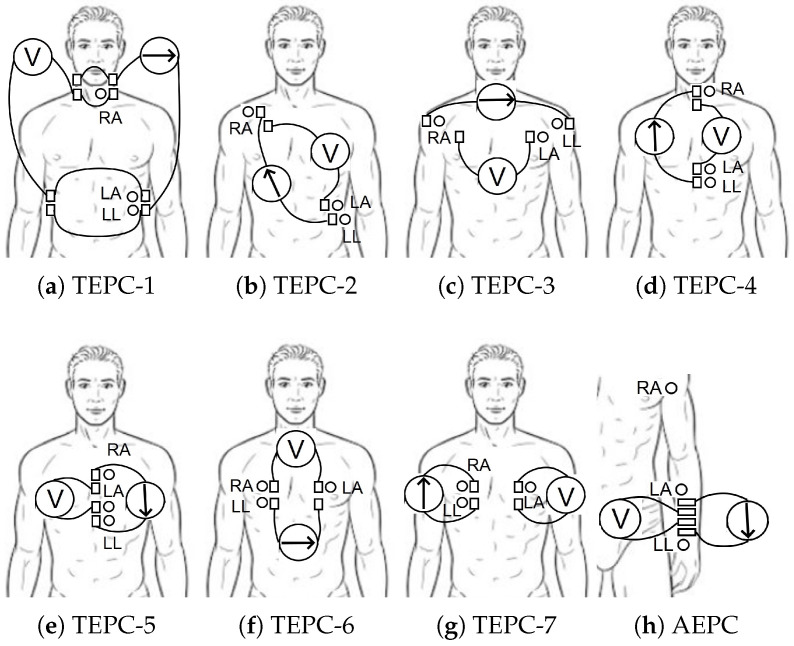
Measurement configuration setups: TEPC-1 to TEPC-7 (where the signal acquiring technique for ICG with excitation (arrow, denoting the direction of current) and measurement (letter V) parts in line with the electrode placement of ECG) are shown (modified from [12]) and AEPC.

The locations of electrodes for ECG signal acquisition were selected based on the ICG electrode placements. More specifically, the ECG electrodes were attached alongside the electrodes of ICG [12]. The electrodes were physically separated for both techniques to avoid the additional electronic circuitry for signal pick-up that is necessary in the case of co-usage. Altogether, eight different EPCs were evaluated for detecting ECG, from which seven were formed on the thorax (TEPC, i.e., thoracic EPC) (TEPC1-7) and one was formed on single arm (AEPC, i.e., arm EPC) (AEPC) (Figure 4).

**Figure 5:**
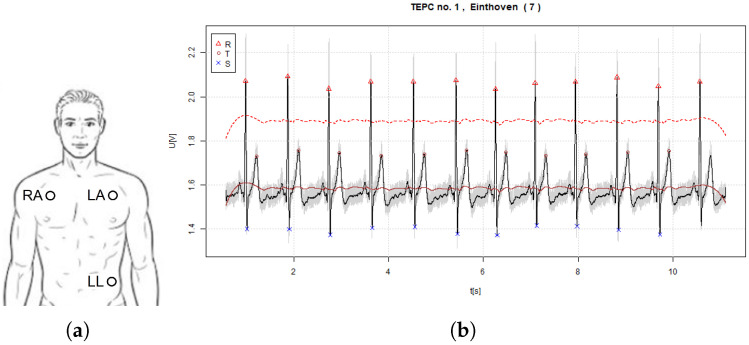
Three-electrode system for monitoring ECG, containing right arm (RA), left arm (LA), and left leg (LL) electrodes in standard positions (**a**) and an example of the acquired signal of ECG (where the continuous red line denotes the baseline and dashed red line 60% threshold level) (**b**).

For reference, in the cases of the same ICG electrode locations, ECG was picked up also with a standard 3-electrode system (where a single lead was considered in signal analysis). In such case, the positive left arm (LA) electrode, right arm negative (RA) electrode, and left leg (LL) electrode were attached with equal distances on the thorax, constituting a standard ECG monitoring approach (Figure 5a). A respectively monitored signal of ECG is visible in Figure 5b.

No measurement experiments were performed in the frame of the current paper. The existing data set of ECG signals, gathered during earlier experimentation, was used. In the reused data set, the signals of ICG and ECG were simultaneously gathered from a variety of locations on the body surface of a single volunteer (37 years of age, slim, healthy) with different EPCs.

In every data log, three consecutively acquired signals of ECG (and ICG at 4 pre-selected excitation frequencies) in the case of every EPC were present. For evaluation, a single log for each EPC was selected fulfilling the criteria of being of relatively comparable quality. In [12], only the signals of ICG were analyzed; so, the current work relies on the previously unanalyzed data.

## 4. Results

**Figure 6:**
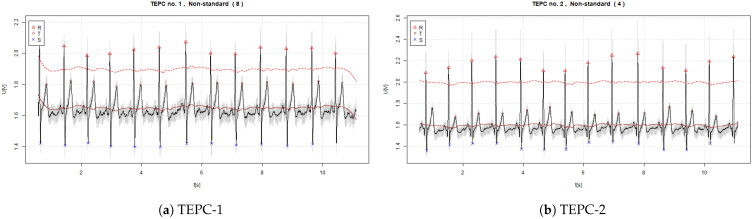

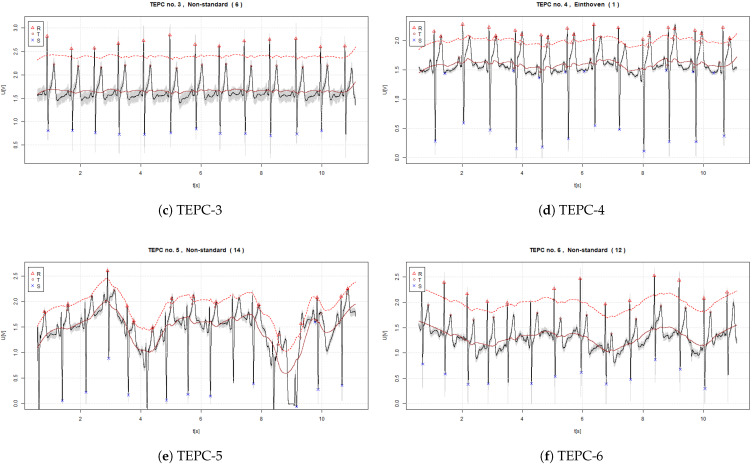
Measured signals of ECG that are acquired by using the TEPC1-6 (where the continuous red line denotes the baseline and dashed red line 60% threshold level).

**Figure 7:**
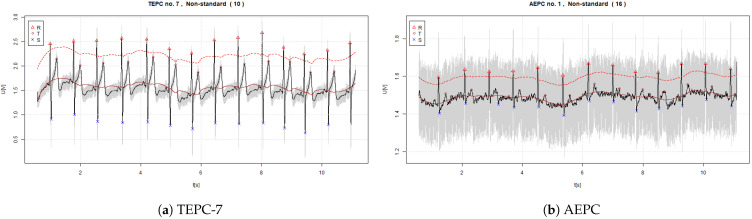
Measured signals of ECG acquired using the TEPC7 and AEPC (where the continuous red line denotes the baseline and dashed red line 60% threshold level).

The signals of ECG with estimated R, S, and T waves that were acquired by using the non-standard EPCs are visible in Figure 6 and Figure 7.

In the graphs, the continuous red line denotes the midpoint (0-point) of the signal. The dashed red line denotes the threshold value of 60% from the 0-point. The legend for determined R, S, and Q waves is shown in each figure.

The quantitative evaluation can be made based on Table 1, where the chosen and pre-defined signal-morphology-related parameters of real ECG signals are shown. The shown result is the median value of three consecutive measurements in the case of all EPCs, except the last row (standard), which is the median value of all measurements, performed with the standard ECG 3-electrode system (where single-lead data were utilized).

**Figure 8:**
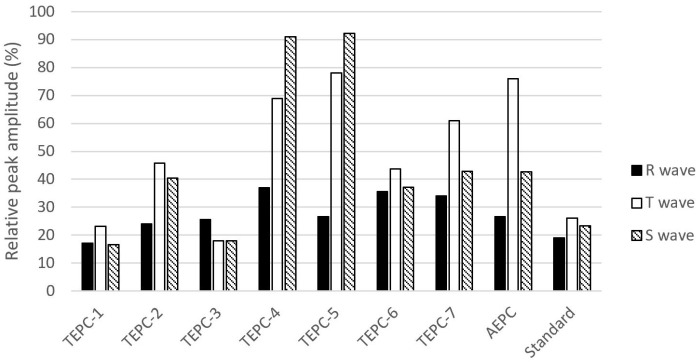
Calculated ratios of differences between the minimum and maximum peak values of R, S, and T waves from the maximum peak values.

The comparison of ratios of calculated differences between the minimum and maximum peak values (in the case of three consecutively measured logs) to the maximum values of R, S, and T waves (relative peak amplitudes) are visible in Figure 8.

The results of applying the proposed approach for distinguishing R waves from large T waves is visible in Table 2. The result is in numerical form, i.e., the calculated width of the wave at 60% threshold level and the areas of the peaks are shown. The results in Table 2 are gained by using the TEPC-3 (the EPC is visible in Figure 4c and the waveform is visible in Figure 6c). The result is shown for six consecutive periods of signal.

**Figure 9:**
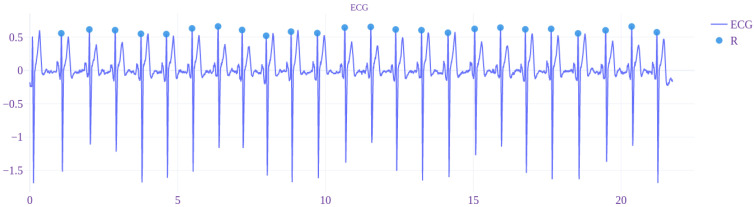
The result of application of the proposed R wave distinguishing algorithm on the signal of ECG that is acquired by using TEPC-4.

**Figure 10:**
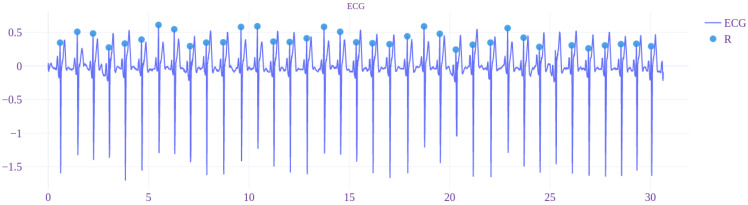
The result of application of the proposed R wave distinguishing algorithm on the signal of ECG that is acquired using TEPC-5.

In Figure 9 and Figure 10, the results of application of the proposed R wave distinguishing algorithm can be seen. Only the two electrocardiograms (acquired by using TEPC-4 and -5), where the phenomenon of the large T wave that is comparable or larger than the R wave appears, are presented. In the example signals, before the application of the proposed algorithm, the removal of 50 Hz interference and the baseline correction is performed, based on the approach described in [49].

## 5. Discussion

The quality estimation of ECG signals can be given based on different signal features. The highly important feature—R wave—which is clinically most used in annotating the ECG signals to determine the HR [29], is one of those. A rough estimation of the signal quality can be made empirically, based on visual impression, given in Table 1 (column: Quality ECG). In defining the quality categories, the proposed levels are based on the same approach as for ICG signals in [12]. However, as in the case of the analyzed ECG signals the R, S, and T waves are visually recognizable in almost 100% of cases, the level 0 (non-recognizable waveform) was omitted. The used categories are 1 (waveform visible but deformed), 2 (reasonable), 3 (strong and clear signal).

A sure indicator of the quality of the ECG signal is the amplitude of R wave—A_Rwave_. Based on this, TEPC-3, -6, and -7 stand out. The same may apply for the negative extremum—the S wave (notable results are provided by TEPC-3, -5, -6, and -7). In the case of T wave, the excessive amplitude may denote a pathological condition and propose a challenge in R wave detection; an example of such a case is TEPC-5.

An issue with the ECG signal can be detected based on its RR interval and corresponding derivatives. Confirmed by Figure 6d,e, the results for R_RRintmaxmin_ in the cases of TEPC-4 and -5 (Table 1) can be recognized as problematic. The determined issue here is the unsuitability of the initial algorithm to differentiate between R and T waves if both waves are of comparable amplitude. The deviation of the data of these EPCs can also be realized in the value of HR, which, in the case of similar log length, is higher than in other cases.

The proposed R and T wave distinguishing approach will provide a solution, indicating the clear difference in the proposed threshold level concerning the width of the peak (Table 2). The peak width of T wave is about 2–3 times larger than for R wave. The difference is smaller in the case of the area calculation. Based on Figure 9 and Figure 10, the performance of the proposed R wave distinguishing algorithm can be evaluated. As no false positives in the example signals were present, the assessment can be concluded as very good. However, the algorithm needs testing on a larger amount of data and variety of signals with inter-individual differences.

The comparison based on wave (R, S, and T) amplitudes was found to provide an effective means for performing SQA. The smaller the ratio of difference between the determined maximum and minimum amplitudes to the maximum peak value, the better the signal quality (Figure 8). Moreover, the remarkably lower sensitivity of R wave can be realized when compared with T and S waves. The median sensitivities for the data, presented in Figure 8, are as follows: 27.3% (R wave); 49% (T wave); 44.9% (S wave). The reason is expected to be the higher variability of R wave among the analyzed signals of ECG.

As can be realized, the quality of ECG signals is largely dependent on the exact positioning of electrodes on the body surface, matching with the similarly appearing phenomena in the case of ICG signals [12]. However, when considering the signals of ICG and ECG, one has to bear in mind the origin of the signal; in the case of ECG, the electrical signal—originating from the sinus node—propagates through the body. Meanwhile, in the case of ICG, the mechanical pumping activity of the heart is detected through determining the respective volume changes in the body by applying external stimulation [50]. It would be interesting to compare the quality estimations of both signals (ICG and ECG) acquired from the joint EPCs. The result is visible in Table 3, indicating TEPC-1 and TEPC-7 as recommendable EPCs for simultaneous detection of ICG and ECG.

From the literature, not many comparable reports of similar approaches for joint acquisition of the signals of ICG and ECG can be found. The proposed solutions are typically based on the classical spot-electrode arrangement based approach [19]), which covers the full thorax, such as in [51]. Concerning more heavily modified EPCs, only a single thoracic area based approach can be found, reported by Hafid et al. [13]. Concerning the wrist, proposals exist in granted patents, e.g., of Apple Inc. (Cupertino, CA, USA) [52], where the acquisition of the signals of ICG and ECG, along with other sensor-fusion-based sensorics, have been proposed. However, as described in the introductory section, for ECG, contact with a finger of the opposite arm is typically required and no exact and variety of EPCs have been shown nor can be found from the literature. To our knowledge, no approaches for simultaneous acquisition of the signals of ICG and ECG from the wrist have been published, revealing the novelty of the current paper.

For ECG signal quality estimation, the result matches nicely with the statistical approach, presented in Figure 8. A contrast appears in the case of AEPC, where the signal of ECG is relatively weak, while the signal of ICG gains the highest grade. From a clinical point of view, the ECG is irreplaceable, providing data that are not possible to gain by other methods (such as ICG, PPG, etc.). So, efforts are required in clever regional signal detection method and capable instrumentation development.

## 6. Limitations of the Current Work

The current work is limited in certain aspects that must be considered to make substantial conclusions. First of all, in determining the most suitable non-standard EPCs, only data acquired from single volunteer were utilized. The results indicate the suitability of the proposed EPCs; however, a larger sample size is needed to verify the results. Moreover, as the EPCs were pre-selected by the authors, based on the experience from practical experimentation, a full mapping of the whole body could reveal more suitable electrode positioning areas.

Secondly, the SQA is performed based on the selected fiducial points of the signal of ECG. The performance could be further improved by attracting more features, maybe also in the frequency domain.

Thirdly, the proposed R wave distinguishing approach and algorithm needs further verification on a larger number of signals. In current research, the performance of the algorithm can be evaluated to be satisfactory but needs comparison with the performance of existing models.

## 7. Conclusions and Future Work

The signals of ECG, acquired from pre-defined EPCs, can all be categorized as conditionally acceptable or acceptable. Based on the visual and statistical assessment, in all of the cases, all of the R, S, and T waves can be detected. However, the possible distinctness of the signals of ECG, manifesting in non-standard acquisition method—where, for example, the domination of T wave over R wave appears—propose a challenge.

The joint acquisition of the signals of ECG and ICG by using the same electrodes (or locations) is highly feasible; however, it is largely dependent on the exact EPC. TEPC-1 and -7 are noted as the most suitable ones, based on the presented data. However, the single-arm-based approach also provides a conditionally acceptable result.

The applied trivial SQA approach was shown to be effective in cases of ECG signals that are acquired using the proposed non-standard EPCs, especially without a pathological or measurement-related artifact. The help of the proposed R and T wave distinguishing approach is evident but needs verification on a larger amount of data.

The current work is expected to be extended by adding a third type of sensor into the composition—the PPG sensor. The PPG sensor can be attached close to the co-utilized electrodes for ECG and ICG signal acquisition, predictably giving valuable supportive data. As a result, based on the outcomes, the design principles of the next-generation Holter monitor can be foreseen. Moreover, as a further development, tailor-made algorithms are needed to distinguish between different pathology-related ECG signal feature deviations (such as short QT, negative T wave, etc.).

## Figures and Tables

**Table 1:** Numerical results of feature extraction. The values of pre-selected features of the measured ECG signals together with quality estimation (1—conditionally acceptable; 2, 3—acceptable).

EPC	A_Rwave_ (V)	A_Twave_ (V)	A_Swave_ (V)	T_RRint_ (s)	R_RRintmaxmin_	HR	Quality ECG
TEPC-1	0.423	0.183	0.266	0.876	1.139	13	3
TEPC-2	0.685	0.169	0.237	0.877	1.159	12	3
TEPC-3	1.204	0.553	0.947	0.889	1.137	12	3
TEPC-4	0.461	0.182	0.213	0.919	4.174	21	2
TEPC-5	0.570	0.373	1.444	1.019	4.830	17	1
TEPC-6	1.137	0.607	0.967	1.297	1.818	12	2
TEPC-7	1.093	0.573	0.797	0.130	1.180	13	3
AEPC	0.174	0.051	0.069	0.864	1.400	14	1
Standard	0.665	0.246	0.368	0.889	1.149	12	3

**Table 2:** Numerical results of applying the proposed approach for distinguishing R waves from large T waves on the ECG signal (based on the waveform acquired using the TEPC-3).

Parameter	1	2	3	4	5	6
R peak width	0.023	0.019	0.018	0.020	0.021	0.024
T peak width	0.070	0.237	0.081	0.078	0.072	0.069
R peak area	0.026	0.018	0.017	0.020	0.023	0.030
T peak area	0.037	0.045	0.044	0.043	0.038	0.035

**Table 3:** Quality estimation comparison of the signals of ICG (0, 1—not acceptable; 2, 3—acceptable [12]) and ECG (1—conditionally acceptable; 2, 3—acceptable) for the same EPCs.

EPC	Quality ICG	Quality ECG
TEPC-1	3	3
TEPC-2	1	3
TEPC-3	1	3
TEPC-4	2	2
TEPC-5	1	1
TEPC-6	0	2
TEPC-7	2	3
AEPC	3	1

## Data Availability

Not applicable.

## Abbreviations

The following abbreviations are used in this manuscript:

EPC	Electrode Placement Configuration
TEPC	Thoracic Electrode Placement Configuration
AEPC	Arm Electrode Placement Configuration
ECG	Electrocardiography
ICG	Impedance Cardiography
HR	Heart Rate
SQA	Signal Quality Assessment
PPG	Photoplethysmography
LVET	Left Ventricular Ejection Time
PEP	Pre-Ejection Period
SNR	Signal-To-Noise Ratio
P-QRS-T	Electrocardiogram Waves
